# CT Imaging Biomarkers in Rhinogenic Contact Point Headache: Quantitative Phenotyping and Diagnostic Correlations

**DOI:** 10.3390/jimaging11100362

**Published:** 2025-10-14

**Authors:** Salvatore Lavalle, Salvatore Ferlito, Jerome Rene Lechien, Mario Lentini, Placido Romeo, Alberto Maria Saibene, Gian Luca Fadda, Antonino Maniaci

**Affiliations:** 1 Department of Medicine and Surgery, School of Medicine, University of Enna “Kore”, 94100 Enna, Italy; salvatore.lavalle@unikore.it (S.L.);; 2 Department of Medical and Surgical Sciences and Advanced Technologies “GF Ingrassia”, ENT Section, University of Catania, 95125 Catania, Italy; ferlito@unict.it; 3 Research Committee, Young Otolaryngologists of the International Federation of Otorhinolaryngological Societies, 70123 Paris, Francealberto.saibene@gmail.com (A.M.S.); 4 Department of Surgery, UMONS Research Institute for Health Sciences and Technology, University of Mons (UMons), 7000 Mons, Belgium; 5 ASP Ragusa-Hospital Giovanni Paolo II, 97100 Ragusa, Italy; 6 Division of Radiology San Marco Hospital, AOU Policlinico “G. Rodolico” San Marco, 95121 Catania, Italy; 7 Department of Otolaryngology-Head and Neck Surgery, University of Milan, 20019 Milan, Italy; 8 Department of Otolaryngology, University of Turin, “San Luigi Gonzaga” Hospital, 10043 Turin, Italy

**Keywords:** rhinogenic contact point headache, computed tomography, imaging biomarkers, phenotyping, quantitative radiology

## Abstract

Rhinogenic contact point headache (RCPH) represents a diagnostic challenge due to different anatomical presentations and unstandardized imaging markers. This prospective multicenter study involving 120 patients aimed to develop and validate a CT-based imaging framework for RCPH diagnosis. High-resolution CT scans were systematically assessed for seven parameters: contact point (CP) type, contact intensity (CI), septal deviation, concha bullosa (CB) morphology, mucosal edema (ME), turbinate hypertrophy (TH), and associated anatomical variants. Results revealed CP-I (37.5%) and CP-II (22.5%) as predominant patterns, with moderate CI (45.8%) and septal deviation > 15° (71.7%) commonly observed. CB was found in 54.2% of patients, primarily bulbous type (26.7%). Interestingly, focal ME at CP was independently associated with greater pain severity in the multivariate model (*p* = 0.003). The framework demonstrated substantial to excellent interobserver reliability (κ = 0.76–0.91), with multivariate analysis identifying moderate–severe CI, focal ME, and specific septal deviation patterns as independent predictors of higher pain scores. Our imaging classification system highlights key radiological biomarkers associated with symptom severity and may facilitate future applications in quantitative imaging, automated phenotyping, and personalized treatment approaches.

## 1. Introduction

Rhinogenic contact point headache (RCPH) is a recognized secondary headache disorder, described in the International Classification of Headache Disorders, that arises from mucosal CP within the nasal cavity—most commonly between the nasal septum and adjacent turbinate or lateral nasal wall—in the absence of active sinonasal inflammation, polyps, or masses [1,2]. Patients typically present facial or periorbital pain that is reproducibly diminished by topical anesthesia put at the contact sites. In addition, the pain is significantly relieved following surgical correction, emphasizing a distinct pathophysiological basis from primary headache syndromes [1]. The role of anatomical variants—such as a deviated nasal septum, septal spurs, or pneumatized middle turbinate (CB)—has been implicated in creating mucosal CP that may activate nociceptive trigeminal pathways [2,3,4]. However, the precise mechanisms linking these structural features to headache generation remain controversial, with debates centering on whether such CP have true etiological relevance or represent incidental findings [5]. Computed tomography (CT) offers highly detailed visualization of the sinonasal anatomy, allowing identification and characterization of CP variations. However, the literature lacks comprehensive quantitative phenotyping of CT biomarkers—such as the degree or angle of septal deviation, surface area of contact, volumetric assessment of turbinate pneumatization, or spatial relationships of mucosal interfaces—and how these imaging metrics correlate with clinical severity, headache phenotype, or treatment response [3,6]. At present, a clinical history, nasal endoscopy with high-resolution CT imaging and detection of mucosal CP along with the response to a local anesthetic (lidocaine) test are used for making the diagnosis of RCPH [1,2,7]. Unfortunately, this method is highly qualitative and operator dependent and is often reduced to descriptive scoring without well-defined quantitative imaging metrics. This hinders the ability of the diagnosis to be reproduced, and an accurate surgical plan may be difficult to model. To avoid these limitations, we outlined a CT-based quantitative framework that furnishes unbiased imaging biomarkers—including CI, septal deviation angle, and ME—for the prediction of disease severity and surgical benefit. Inclusion of these variables in pre-operative evaluation therefore may help to better stratify patients for surgery, tailor the amount of mucosal correction and avoid unnecessary intervention. Furthermore, while surgical intervention—typically endoscopic removal or correction of mucosal contacts—has demonstrated superiority over purely medical management in reducing headache severity, duration, and frequency (as measured by the Visual Analog Scale (VAS), attack duration, frequency, and by disability scores such as the Migraine Disability Assessment Scale (MIDAS)), little is known about which CT-derived anatomical subtypes predict favorable outcomes [1,7]. Therefore, this study aims to fill these gaps by implementing a rigorous quantitative CT-imaging protocol to phenotype intranasal CP in RCPH, and to assess correlations between specific imaging biomarkers and both diagnostic accuracy and post-intervention clinical response.

Our study introduced a standardized quantitative framework for the evaluation of RCPH, combining multiple CT-based anatomical parameters, such as contact intensity (CI), septal deviation angle, and mucosal edema (ME) pattern, within a unified analytical model. This integrated approach may propose a methodological advance over previous qualitative descriptions, providing a reproducible basis for future AI-driven imaging studies.

The objectives of this study were to (i) propose a standardized definition for assessing RCPH using CI, septal deviation angle and ME pattern in an objective quantitative manner; (ii) demonstrate the associations between imaging biomarkers and patient-rated symptom burden by multivariate and cluster analysis; (iii) provide reproducible diagnostic insights; and (iv) illustrate how these novel radiological metrics could be used as potential tools for clinical decision making or surgical strategy by allowing calculation of RCPH throughout preoperative evaluation. The following parts of this paper were set up explaining the study design, patient inclusion and imaging analysis: a report of the main results and a quantitative evaluation of radiological parameters in relation to clinical severity; discussing the clinical relevance, methodological implications and limitations of adopting standardized imaging protocols for RCPH; and, finally, offering conclusory remarks and perspectives on further prospects for conducting studies into the routine standardization of imaging methods used to assess RCPH.

## 2. Materials and Methods

### 2.1. Study Design and Ethical Considerations

This was a prospective multicenter study conducted at the departments of otolaryngology and radiology, in accordance with the principles of the Declaration of Helsinki. The study was reported following the Strengthening the Reporting of Observational Studies in Epidemiology (STROBE) guidelines to ensure methodological transparency and reproducibility [8]. Ethical approval was obtained from the Ethics Committee of San Luigi Gonzaga Hospital (protocol code no. 44/2022, 28 March 2022). All data were collected from anonymized imaging and clinical records. No personally identifiable information was stored or shared. Patient consent was waived due to the retrospective and non-interventional nature of the study, as approved by the ethics committee. Written informed consent was obtained from all participants, authorizing the use of anonymized data for research purposes.

### 2.2. Study Population

A total of 120 patients presenting with clinical suspicion of RCPH were enrolled between [April, 2022] and [August, 2025] and screened for eligibility. Inclusion criteria were (1) diagnosis of RCPH according to the International Classification of Headache Disorders criteria (ICHD-3); (2) presence of mucosal CP on nasal endoscopy or CT imaging in the absence of acute sinusitis, nasal polyposis, or tumors; (3) age ≥ 20 years; and (4) exhibiting a positive response to a lidocaine test, where pain relief was achieved upon lidocaine application to the nasal cavity.

Exclusion criteria were as follows:

- Less than one year of consecutive clinical and diagnostic follow-up.
- Presence of comorbid conditions including allergies, other sinonasal disorders, migraines, cluster headaches, ophthalmologic or vascular disorders, hypertension, pregnancy, or temporomandibular joint disorders.
- A history of previous sinonasal surgeries.

The exclusion of patients with allergic rhinitis or chronic sinonasal inflammation was intentional so as to minimize potential confounding factors. Allergic ME and TH may transiently alter nasal anatomy and airflow, thereby influencing CI and symptom expression independent of true structural contact mechanisms. Similarly, patients with migraine, tension-type, or vascular headaches were excluded to isolate the mechanical etiology of RCPH and ensure homogeneity of the study cohort.

### 2.3. Clinical Assessment

All patients underwent standardized clinical evaluation, including detailed headache history (onset, duration, frequency, and laterality). Headache severity was quantified using a VAS (0–10). Topical nasal anesthesia (cotton pledget test) was applied to confirm symptom relief attributable to CP.

### 2.4. Imaging Protocol and Quantitative Analysis

#### 2.4.1. Image Acquisition Protocol

High-resolution CT scans of the paranasal sinuses were obtained (slice thickness: 0.5–1 mm, bone window algorithm) in axial, coronal, and sagittal planes. Imaging was reviewed independently by two blinded radiologists and one otolaryngologist.

#### 2.4.2. Quantitative Parameters

CT scans were systematically evaluated for seven parameters, as follows:

1. Contact point type: Classified as CP-I (septum-middle turbinate), CP-II (septum-inferior turbinate), CP-III (septum-lateral wall), and CP-IV (other variants).
2. Contact intensity: Categorized as mild, moderate, or severe based on the degree of mucosal compression.
3. Septal deviation: Measured at the point of maximum deviation and classified as SD-A (<15°) or SD-B/SD-C (>15°).
4. CB morphology: Categorized as absent, bulbous, lamellar, or extensive types.
5. Mucosal edema: Assessed as ME-1 (focal edema) or ME-2 (diffuse edema) at CP.
6. TH: Classified as absent, mild, moderate, or severe.
7. Associated anatomical variants: Including paradoxical middle turbinate, Haller cells, pneumatized uncinate process, etc.

Additional quantitative measurements included the following:

- Surface area of mucosal contact (mm^2^), calculated using multiplanar reconstruction.
- Turbinate pneumatization volume (mm^3^) when applicable.
- Laterality index, defined as unilateral vs. bilateral involvement.

#### 2.4.3. Inter-Observer Agreement

Inter-observer agreement was assessed using Cohen’s kappa and intraclass correlation coefficients (ICC) for all imaging parameters.

### 2.5. Treatment and Follow-Up

This study focused primarily on imaging biomarkers and their correlation with clinical parameters rather than treatment outcomes. However, baseline treatment data were collected for future longitudinal analysis. Patients with confirmed RCPH received either conservative medical therapy (analgesics, nasal decongestants, intranasal corticosteroids) or endoscopic surgical correction of CP based on standard clinical practice and patient preference. Initial baseline pain scores were documented using the Visual Analog Scale (VAS) and used to correlate with radiological findings.

### 2.6. Statistical Analysis

Data were analyzed using Jamovi software (2.6.5). Continuous variables were expressed as mean ± standard deviation (SD) or median (interquartile range (IQR)), while categorical variables were summarized as frequencies and percentages.

Between-group comparisons were performed using Student’s *t*-test for normally distributed continuous variables or Mann–Whitney U test for non-parametric data. ANOVA with post-hoc Tukey tests was used to assess differences in VAS scores across CI and CB subtypes. χ^2^ or Fisher’s exact tests were applied for categorical comparisons. Correlations between continuous CT biomarkers (e.g., septal deviation angle, ME, CI) and clinical severity (VAS) were tested using Pearson’s correlation for normally distributed data or Spearman’s rank correlation otherwise. Multivariate linear regression models were then built to identify independent imaging predictors of higher pain scores, with statistical significance set at *p* < 0.05. Interobserver reliability was quantified using kappa statistics, with values interpreted as follows: κ < 0.40 (poor), κ = 0.40–0.59 (fair), κ = 0.60–0.75 (good), and κ > 0.75 (excellent). A *p*-value < 0.05 was considered statistically significant.

Cluster analysis was used to group patients who shared similar imaging and clinical characteristics, helping us identify typical “profiles” of RCPH. Three main clusters emerged, as follows:

- Cluster 1 (mild): Patients with light mucosal contact and minimal structural deviation, reporting the lowest pain scores.
- Cluster 2 (moderate): Patients with moderate CI and ME-1, showing intermediate pain levels.
- Cluster 3 (severe): Patients with strong mucosal compression and ME-2, experiencing the highest pain severity.

## 3. Results

### 3.1. Patient Demographics and Clinical Characteristics

We analyzed 120 consecutive patients with RCPH with complete clinical–radiological data (Table 1).

### 3.2. Radiological Phenotype Distribution

**Figure 1:**
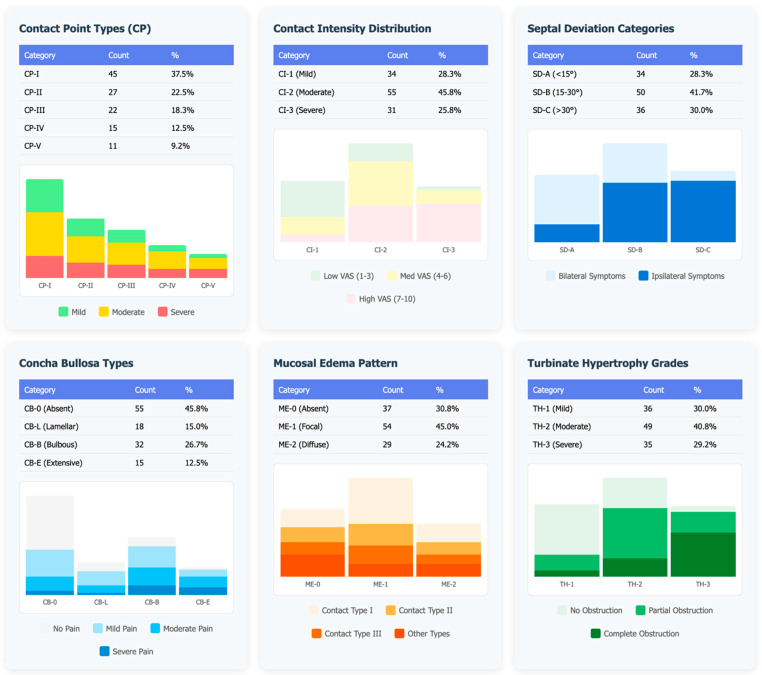
Graphical representation of radiological phenotype distribution.

CT imaging showed a heterogeneous distribution of CP phenotypes. CP-I was the most frequent configuration (39/120, 32.5%), followed by CP-II (24/120, 20.0%) and CP-IV (23/120, 19.2%), whereas CP-III and CP-V accounted for 21/120 (17.5%) and 13/120 (10.8%), respectively (Figure 1).

CI was predominantly moderate (53/120, 44.2%), with mild and severe grades in 35/120 (29.2%) and 32/120 (26.7%), respectively. Septal deviation categories were distributed as SD-A 32/120 (26.7%), SD-B 46/120 (38.3%), and SD-C 42/120 (35.0%). CB was absent in 61/120 (50.8%), while bulbous (CB-B) was the most common subtype among present CB (39/120, 32.5%), followed by lamellar (CB-L 16/120, 13.3%) and extensive (CB-E 4/120, 3.3%). ME at the CP was most often focal (ME-1 65/120, 54.2%), whereas it was absent in 29/120 (24.2%) and diffuse in 26/120 (21.7%). TH was grade 2 in 48/120 (40.0%) and grade 3 in 44/120 (36.7%), exceeding grade 1 (28/120, 23.3%). AV were frequent: paradoxical middle turbinate 31/120 (25.8%), Haller cells 28/120 (23.3%), Onodi cells 13/120 (10.8%), agger nasi variants 16/120 (13.3%); 20/120 (16.7%) had no variant.

### 3.3. Correlation Between Imaging Biomarkers and Pain Severity

**Figure 2:**
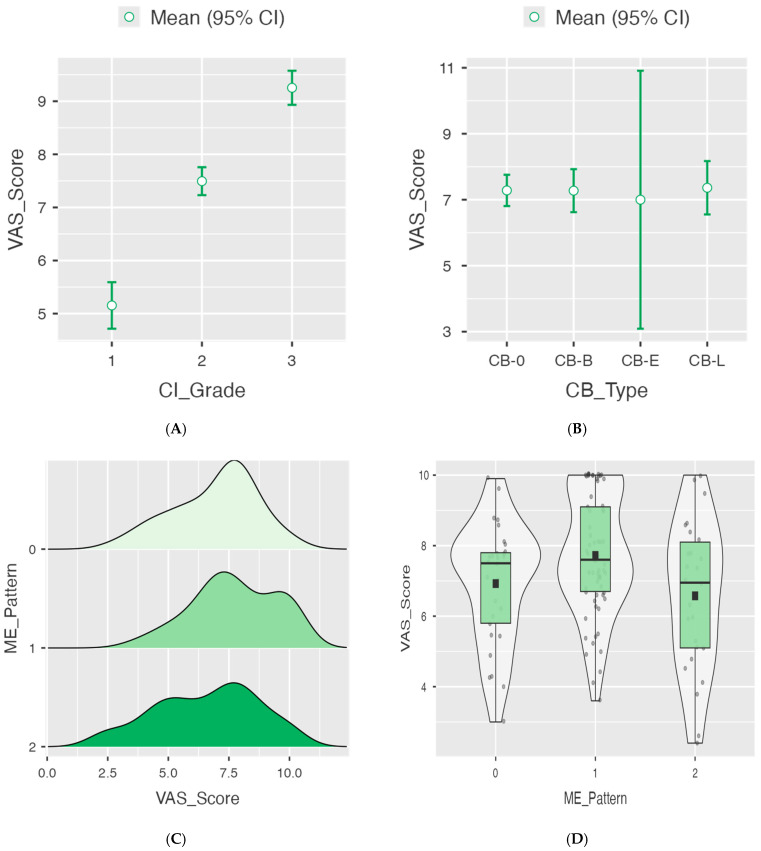
Clinical–radiological correlations between imaging biomarkers and headache severity (VAS) in RCPH. (**A**) Boxplots showing mean VAS values increasing significantly with CI grades 1–3 (*p* < 0.001, one-way ANOVA). (**B**) Comparison of VAS across CB subtypes, with no significant difference (*p* = 0.993). (**C**) Density plots illustrating the distribution of VAS according to ME categories; patients with ME-1 showed higher VAS scores than ME-0 (absent) or ME-2 (diffuse). (**D**) Violin plots summarizing VAS variability across ME patterns, with median values and 95% confidence intervals. All data are derived from the total cohort (*n* = 120); analyses performed using Welch ANOVA and Tukey post-hoc tests. Error bars represent 95% confidence intervals. Statistical significance was defined as *p* < 0.05. Open green circles indicate group means (95% CI). Black squares represent mean values within each violin plot.

Pain severity (VAS) differed markedly across CI grades (Welch ANOVA F = 118, df1 = 2, df2 = 68.1, *p* < 0.001) (Figure 2A–D).

Post-hoc Tukey contrasts showed higher VAS for severe vs. mild (mean difference 4.10, *p* < 0.001) and severe vs. moderate (mean difference 1.76, *p* < 0.001), and for moderate vs. mild (mean difference 2.34, *p* < 0.001). Assumption checks supported model adequacy (Shapiro–Wilk *p* = 0.301; Levene *p* = 0.075) (Table 2).

### 3.4. Multivariate Predictors of Clinical Severity

Although the univariate analysis for ME (Table 3) did not reach statistical significance, the multivariate regression model confirmed focal ME (ME-1) as an independent predictor of higher VAS scores (*p* = 0.003) after adjusting for CI and septal deviation angle. Conversely, VAS did not differ by CB subtype (Welch ANOVA F = 0.0289, df1 = 3, df2 = 13.1, *p* = 0.993; all Tukey contrasts non-significant; Shapiro–Wilk *p* = 0.006; Levene *p* = 0.365) (Figure 2). Descriptively, mean VAS was 7.72 ± 1.71 in ME-1, 6.92 ± 1.72 in ME-0, and 6.58 ± 2.12 in ME-2 (one-way ANOVA *p* = 0.566) (Figure 2). In a simple linear model treating ME as ordinal, the association with VAS was not statistically significant (β = −0.145, SE = 0.252, *p* = 0.566; model R = 0.053, R^2^ = 0.003) (Table 3).

**Figure 3:**
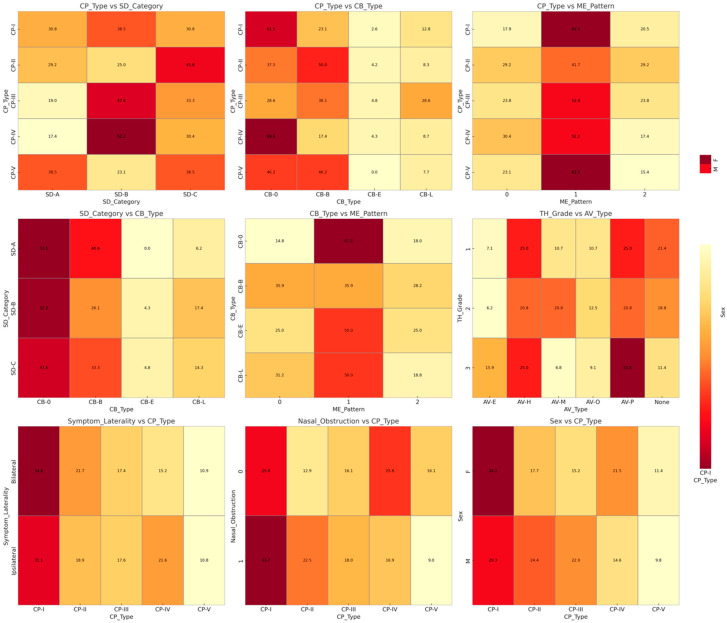
Correlation matrix. Color-coded Spearman correlation coefficients (ρ) display the co-occurrence between CT-based variables: CI, ME, septal deviation, CB, TH, and VAS. Darker colors represent stronger positive correlations (*p* < 0.05).

Co-occurrence maps illustrated how structural variants clustered within subjects’ CP phenotypes tended to appear with moderate–severe septal deviation (SD-B/C), and these deviations frequently co-existed with bulbous or lamellar CB. TH often accompanied AV—most notably paradoxical middle turbinate—while symptom laterality mirrored the side of deviation and the dominant CP (Figure 3). To illustrate these results more intuitively, a co-occurrence (correlation) matrix visualizes how various anatomic and imaging features tend towards being present in the same patients. Greater CI and pain scores appear to correspond more commonly with bulbous or lamellar CB in patients who have more severe septal deviation. Practically, the matrix provides information on which combinations of structures are likely to generate clinically relevant contacts.

Spearman analysis highlighted strong positive correlation between CI grade and VAS (ρ = 0.844, *p* < 0.001) and moderate associations between the Imaging Severity Index (ISI) (ISI) and SD angle (ρ = 0.630, *p* < 0.001), ME pattern (ρ = 0.567, *p* < 0.001), CI grade (ρ = 0.450, *p* < 0.001), and VAS (ρ = 0.352, *p* < 0.001). Other pairwise correlations were weak and non-significant (Table 4).

### 3.5. Cluster-Based Patient Stratification

**Figure 4:**
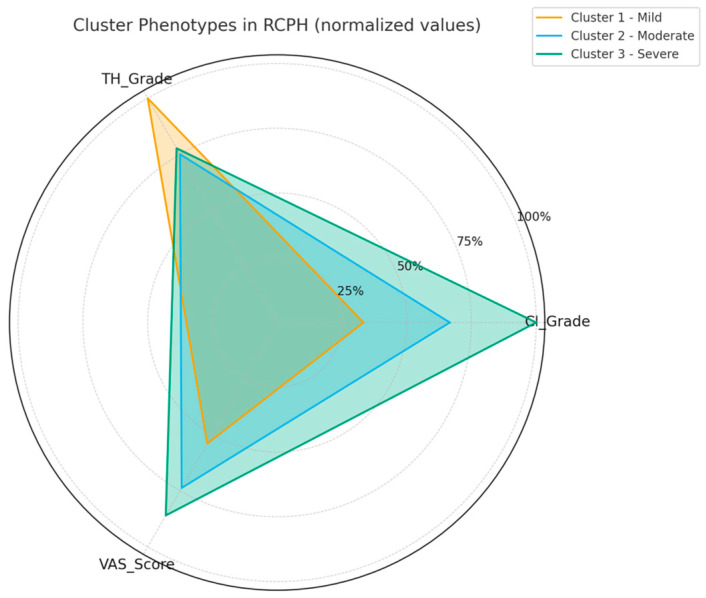
Cluster-based phenotyping of RCPH patients. Radar plots summarize the average values of imaging biomarkers (CI, ME, SD) and clinical VAS for each identified cluster. (1) Cluster 1 (mild): minimal contact and lowest VAS. (2) Cluster 2 (moderate): moderate CI and focal ME. (3) Cluster 3 (severe): marked CI and diffuse ME with highest VAS. The analysis illustrates how imaging severity scales with symptom intensity.

Cluster analysis identified three phenotypes of RCPH. Cluster 1 (mild, *n* = 1) showed CI grade 1, absence of CB and ME, but severe TH (grade 3), with a VAS of 5.4. Cluster 2 (moderate, *n* = 12) included patients with CI grade 2, SD-C, bulbous CB, focal ME (ME-1), and moderate TH (mean 2.25); mean VAS was 7.4 ± 0.6, significantly higher than grade 1 (*p* < 0.001) but lower than grade 3 (*p* < 0.001). Cluster 3 (severe, *n* = 6) presented CI grade 3, SD-C, bulbous/lamellar CB, diffuse ME (ME-2), and TH grade 2.3, with the highest VAS (8.6 ± 0.9, *p* < 0.001 vs. clusters 1–2). The radar plot (Figure 4) illustrates this gradient, confirming a strong correlation between CI and VAS (ρ = 0.844, *p* < 0.001) and between ME and VAS (ρ = 0.567, *p* < 0.001), while TH showed weaker association (*p* = 0.664) (Figure 4).

These findings highlight CI and ME as the main imaging predictors of clinical severity.

## 4. Discussion

The present multicenter prospective study established and validated a CT-based methodology for quantitative phenotyping of RCPH, offering new insight into the association between anatomical variations, imaging biomarkers and clinical severity. To ensure that headache symptoms were attributable to anatomical contact mechanisms, we excluded patients with allergic or inflammatory sinonasal disorders, which can alter mucosal volume and bias CT-based quantification. We found that CI and ME-1 are the best predictors of headache severity, while other variants, such as CB subtypes, should not weigh as heavily on the clinical decision-making process. The statistical consistency across *t*-tests, ANOVA, and regression models supports the robustness of the imaging biomarkers identified. Our findings are consistent with and extend earlier work indicating that CP between the nasal septum and its surrounding structures can cause nociceptive excitement by inducing trigeminal input [9,10]. La Mantia et al. showed that surgical treatment offered better relief compared with medical treatment [11] and our study extends these observations by providing insight into which CT-derived dimensions correlate best with symptomatology. Moderate-to-severe CI and the finding of edema were independently associated with increased VAS scores, stressing the need to detect and measure mucosal contact. The correlation of septal deviation is a curious one. Although septal deviation is often dismissed as an incidental finding by some authors [12], according to our results, deviations > 15° are more likely to be graded higher for CI and accompanied by AV such as paradoxical middle turbinate or Haller cells. This indicates that septal deviation represents a structural initiator for clinical-level contact establishment. Likewise, bullosa was found in most of our cohort but its subtypes did not significantly contribute to the pain intensity, supporting further studies showing that CB seems alone to be inadequate for RCPH pathogenesis [13]. It is of note that, interestingly, our findings underline the predictive capability for mucosal edema at the CP. This is consistent with histopathologic and neurochemical investigations that have shown how mucosal contact can evoke local neurogenic inflammation through Substance P- and CGRP-mediated mechanisms to facilitate nociceptive signaling [14,15]. This process may be the reason that ME-1 became a strong imaging biomarker of pain intensity. The significance of this study is based on the quantitative imaging methodology used to eliminate subjective criteria in detection and present reproducible measurements. Previously published systematic reviews have documented the heterogeneity of RCPH diagnostic criteria and surgical results [16]. Our approach of standardizing imaging parameters, including CI grade, septal deviation angle, and CSA, brings the possibility for multicenter data harmonization and incorporation into the future with AI-driven image analysis. However, there are a few limitations which should be considered. First, this is a prospective study, but the observation time was short and long-term treatment effects were not systematically evaluated. Second, functional parameters, such as nasal airflow dynamics and patient-reported outcomes other than VAS, were not included in detail. Third, though interobserver agreement was excellent, the framework will need external validation studies in larger populations of patients, for example the pediatric and old population, where there are changes in anatomical variations. Observational design cannot completely control for confounding factors, including undiagnosed migraine overlap, which is a substantial diagnostic problem in this area.

Lastly, although no new imaging technology was developed, the main methodological contribution of this study lies in the quantitative standardization of CT-derived biomarkers for RCPH. By merging anatomical and clinical variables into a single predictive model, our framework enables objective comparison across centers and offers a foundation for automated image-based diagnostic tools.

## 5. Conclusions

In our study, CI and focal mucosal edema were the most clinically relevant CT imaging biomarkers in RCPH, strongly correlating with pain severity. Anatomical variations, such as CB subtypes, may also have less direct impact on headache severity. By defining a reproducible quantitative imaging workflow, we provide a potential platform to enhance diagnostic accuracy, direct surgical intervention and treatment stratification. Future research should focus on incorporating quantitative radiology, radiomics and AI-driven phenatology with clinical outcomes for the purpose of patient specific treatment planning. More extended prospective studies are therefore necessary to confirm these biomarkers’ validity and their predictive value in predicting surgical outcomes. Our study may provide a reproducible methodological basis for integrating radiological and clinical data in RCPH, paving the way for future prospective validation and AI implementation.

## Figures and Tables

**Table 1:** Descriptive table summarizing data categorical, nominal and continuous information. * statistical significant.

Variable	Category	N (%)	Mean VAS ± SD	Range	*p*-Value
Age (years)	–	–	42.3 ± 8.2	29–56	–
Sex	Female	79 (65.8)	–	–	–
Male	41 (34.2)	–	–	–	
Contact intensity (CI)					
Grade 1 (mild)		5.15 ± 1.28	5.2	2.4–7.3	<0.001 *
Grade 2 (moderate)		7.49 ± 0.95	7.6	5.1–9.9	<0.001 *
Grade 3 (severe)		9.25 ± 0.89	9.9	7.4–10.0	Ref
Concha bullosa (CB)					
Absent (CB-0)		7.28 ± 1.85	7.3	2.4–10.0	0.993
Bulbous (CB-B)		7.27 ± 2.01	7.5	2.6–10.0	
Extensive (CB-E)		7.00 ± 2.46	6.8	4.5–9.9	
Lamellar (CB-L)		7.36 ± 1.52	7.6	4.3–10.0	
Mucosal edema (ME)					
ME-0 (absent)		6.92 ± 1.72	7.5	3.0–9.9	0.566
ME-1 (focal)		7.72 ± 1.71	7.6	3.6–10.0	Ref
ME-2 (diffuse)		6.58 ± 2.12	6.95	–2.4–10.0	
Accessory variants (AVs)			–	–	–
Agger nasi (AV-E)	12 (10.0)				
Haller cells (AV-H)	28 (23.3)	–	–	–	
Onodi cells (AV-O)	13 (10.8)	–	–	–	
Paradoxical MT (AV-P)	31 (25.8)	–	–	–	
Miscellaneous (AV-M)	16 (13.3)	–	–	–	
None	20 (16.7)	–	–	–	

**Table 2:** ANOVA outcomes for CI and CB type.

ANOVA results for VAS according to CI Grade.		
Comparison	Mean difference	*p*-value
Mild vs. moderate	−2.34	<0.001
Mild vs. severe	−4.1	<0.001
Moderate vs. severe	−1.76	<0.001
ANOVA for VAS according to CB type.		
Comparison	Mean difference	*p*-value
CB-0 vs. CB-B	0.00761	-
CB-0 vs. CB-E	0.282	0.991
CB-0 vs. CB-L	−0.0805	0.999
CB-B vs. CB-E	0.274	0.993
CB-B vs. CB-L	−0.0881	0.999
CB-E vs. CB-L	−0.3625	0.986

**Table 3:** Linear regression model for VAS as the dependent variable and ME pattern as the predictor.

Predictor	Estimate	SE	95% CI Lower	95% CI Upper	t	*p*-Value
Intercept	7.422	0.299	6.83	8.014	24.825	<0.001
ME pattern	−0.145	0.252	−0.644	0.354	−0.575	0.566

**Table 4:** Correlation of radiological biomarkers and clinical outcomes.

Variable	CI_Grade	SD_Angle	ME_Pattern	TH_Grade	VAS_Score	Imaging_Severity_Index
CI_Grade	—					
SD_Angle	−0.077 (*p* = 0.406)	—				
ME_Pattern	−0.052 (*p* = 0.572)	0.121 (*p* = 0.189)	—			
TH_Grade	0.121 (*p* = 0.188)	−0.078 (*p* = 0.396)	−0.154 (*p* = 0.093)	—		
VAS_Score	0.844 (*p* < 0.001)	−0.133 (*p* = 0.147)	−0.036 (*p* = 0.697)	0.040 (*p* = 0.664)	—	
Imaging_Severity_Index	0.450 (*p* < 0.001)	0.630 (*p* < 0.001)	0.567 (*p* < 0.001)	−0.081 (*p* = 0.380)	0.352 (*p* < 0.001)	—

## Data Availability

The data presented in this study are available on reasonable request from the corresponding author. The data are not publicly available due to privacy and ethical restrictions.

## Abbreviations

The following abbreviations are used in this manuscript:

RCPH	Rhinogenic contact point headache
CT	Computed tomography
VAS	Visual Analog Scale
CP	Contact point
CI	Contact intensity
CB	Concha bullosa
ME	Mucosal edema
TH	Turbinate hypertrophy
AV	Accessory variant
ICHD-3	International Classification of Headache Disorders, 3rd edition
STROBE	Strengthening the Reporting of Observational Studies in Epidemiology
ICC	Intraclass correlation coefficient
ANOVA	Analysis of variance
ISI	Imaging Severity Index
MIDAS	Migraine Disability Assessment Scale

## References

[1] La Mantia I., Grillo C., Andaloro C. (2018). Rhinogenic Contact Point Headache: Surgical Treatment Versus Medical Treatment. J. Craniofac. Surg..

[2] Lavalle S., Pace A., Magliulo G., Lentini M., Lechien J.R., Calvo-Henriquez C., Parisi F.M., Iannella G., Maniaci A., Messineo D. (2025). Impact of Nasal Anatomical Variation Subtype on Surgical Outcomes for Rhinogenic Contact Point Headache. Diagnostics.

[3] Swain S.K., Dubey D. (2018). Anatomical Variations of Nose Causing Rhinogenic Contact Point Headache-A Study at a Tertiary Care Hospital. Pol. Ann. Med..

[4] Swain S.K., Mohanty S., Sahu M.C. (2022). Rhinogenic Contact Point Headache in Pediatric Age Group. Int. J. Contemp. Pediatr..

[5] Bernichi J.V. (2015). Rhinogenic and Sinus Headache-Literature Review. Acta Otorhinolaryngol. Ital..

[6] Patel Z.M., Kennedy D.W., Setzen M., Poetker D.M., DelGaudio J.M. (2013). “Sinus Headache”: Rhinogenic Headache or Migraine? An Evidence-Based Guide to Diagnosis and Treatment. Int. Forum Allergy Rhinol..

[7] Maniaci A., Merlino F., Cocuzza S., Iannella G., Vicini C., Cammaroto G., Lechien J.R., Calvo-Henriquez C., La Mantia I. (2021). Endoscopic Surgical Treatment for Rhinogenic Contact Point Headache: Systematic Review and Meta-Analysis. Eur. Arch. Otorhinolaryngol..

[8] von Elm E., Altman D.G., Egger M., Pocock S.J., Gøtzsche P.C., Vandenbroucke J.P., STROBE Initiative (2008). The Strengthening the Reporting of Observational Studies in Epidemiology (STROBE) Statement: Guidelines for Reporting Observational Studies. J. Clin. Epidemiol..

[9] Welge-Luessen A., Hauser R., Schmid N., Kappos L., Probst R. (2003). Endonasal Surgery for Contact Point Headaches: A 10-Year Longitudinal Study. Laryngoscope.

[10] Clerico D.M. (1996). Pneumatized Superior Turbinate as a Cause of Referred Migraine Headache. Laryngoscope.

[11] Periƒá A., Baletiƒá N., Sotiroviƒá J. (2016). Surgical Treatment of Rhinogenic Contact Point Headache: An Experience from a Tertiary Care Hospital. Acta Otorhinolaryngol. Ital..

[12] Behrbohm H., Tardy M.E. (1991). Contact Point Headaches: Clinical Manifestations and Therapeutic Results. HNO.

[13] Bektas D., Alioglu Z., Akyol N., Ural A., Bahadir O., Caylan R. (2011). Surgical Outcomes for Rhinogenic Contact Point Headaches. Med. Princ. Pract..

[14] Maniaci A., Lechien J.R., Calvo-Henriquez C., Iannella G., Leigh S., Ingrassia A., Merlino F., Bannò V., Cocuzza S., La Mantia I. (2022). Long-Term Stability of Outcomes of Endoscopic Surgery for Rhinogenic Contact Point Headache (Sluder’s Neuralgia). Am. J. Otolaryngol..

[15] Eross E.J., Dodick D.W., Eross M.D. (2007). The Sinus, Allergy and Migraine Study (SAMS). Headache.

[16] Farmer R.L., Garg R.K., Afifi A.M. (2018). Can Functional Nasal Surgery Treat Chronic Headaches? A Systematic Review. Plast. Reconstr. Surg..

